# Image-Based Machine Learning for Predicting Acceptability Limits in Frozen Pizza Shelf Life

**DOI:** 10.3390/foods15081348

**Published:** 2026-04-13

**Authors:** Marika Valentino, Giulia Varutti, Sylvio Barbon Júnior, Maria Cristina Nicoli

**Affiliations:** 1 Department of Agricultural, Food, Environmental and Animal Sciences, University of Udine, Via Sondrio 2/A, 33100 Udine, Italy; marikavalentino@uniud.it (M.V.); giulia.varutti@uniud.it (G.V.); mariacristina.nicoli@uniud.it (M.C.N.); 2 Department of Engineering and Architecture, University of Trieste, Via Alfonso Valerio 6/1, 34127 Trieste, Italy

**Keywords:** color saturation, image-processing, logistic regression classifier, non-destructive method, quality decay

## Abstract

Shelf life of frozen foods is intrinsically linked to consumer sensory acceptability. However, quantifying the synergistic impact of extended storage and variable thermal cycles on perception remains challenging. This study proposes a non-destructive image-based approach for estimating the acceptability of frozen pizza using a machine learning model and identifying tomato sauce degradation as indicator of product quality decay. Qualitative consumer feedback (90%) identified tomato sauce saturation as the primary driver of visual rejection. Image processing pipeline was developed to isolate the sauce region from each sample for further color extraction (saturation in the HSV color space). A second-degree polynomial regression model was used to describe the saturation trend over time and, in parallel, a logistic regression classifier was trained to predict binary consumer acceptability based on both saturation and storage duration. The models were evaluated using frozen pizzas (−12 and −18 °C) for up to 200 days. The regression model achieved an R^2^ of 0.68 and an RMSE of 12.8, while the classifier attained an accuracy of 88.2% and an AUC of 0.93. The resulting framework enables early, non-invasive estimation of product acceptability and shows strong potential for practical application in shelf life studies within the frozen food industry.

## 1. Introduction

Accurate prediction of food shelf life is essential for supporting decision-making in food production, processing, storage, distribution, and consumption [1]. Primary shelf life is defined as the period during which a food product maintains an acceptable level of quality under specified storage conditions, remaining suitable for consumption until it reaches a certain quality threshold, often referred to as the acceptability limit [2]. Predicting the acceptability limit is crucial to ensure food quality and consumer satisfaction. Frozen foods are generally microbiologically stable, which results in an extended shelf life compared to refrigerated or fresh products. Nevertheless, quality deterioration may still occur during storage as a consequence of chemical and physical reactions [3]. In these products, the end of shelf life is generally determined by a loss of sensory quality, making consumer perception a decisive factor in defining product acceptability. Consequently, the identification of the acceptability limit for frozen foods is strongly dependent on consumer evaluation and sensory perception [4]. Traditional shelf life assessment is based on the identification of an early quality indicator that reflects the product’s deterioration over storage time and defining its acceptability limit by sensory acceptability tests. By modeling the kinetics of this quality descriptor over time, it is possible to estimate the product’s shelf life [5]. Although effective, these methods are destructive, resource-intensive, require time-consuming sensory analyses, and they often assume constant storage conditions and packaging properties, which may not reflect real-life distribution and storage variability [6,7]. To overcome these limitations, artificial intelligence (AI) has emerged as a promising tool for predicting food shelf life. AI application in predicting shelf life is a paradigm shift from traditional methods, with more accurate and real-time predictions. Through machine learning (ML), a subset of AI, it is possible to evaluate large datasets from non-destructive measurements and find patterns that may be hard to perceive with more traditional models [8]. In recent years, AI has been successfully applied for shelf life prediction of perishable products, such as fruits and vegetables, where spoilage occurs rapidly due to microbial growth or ripening processes [9,10,11,12,13,14]. In particular, ML combined with image analysis has been widely applied to evaluate product quality and predict shelf life [15,16,17]. These studies typically rely on visual features, including surface defects, color intensity, and texture, demonstrating that image-derived features can effectively predict product quality and shelf life in a rapid, low-cost, and non-destructive manner [18]. Only limited research has extended this approach to frozen foods. Recent work has demonstrated the feasibility of combining computer vision and machine learning to monitor quality changes in frozen products, such as frozen date fruits and frozen-thawed shrimps [19,20]. However, these applications mainly concern single-component foods characterized by relatively homogeneous surfaces, where visual feature extraction is less affected by structural variability. In contrast, multi-component frozen foods represent a significantly more complex scenario. The presence of overlapping ingredients generates spatial heterogeneity and visual noise, complicating the identification of reliable quality markers. Moreover, while image-based methods can monitor visual features, their correlation with consumer acceptability, which is a key determinant of frozen food shelf life, remains limited.

Based on these considerations, the aim of this study was to develop a non-destructive, image-based machine learning approach to predict visual consumer acceptability of frozen pizzas, a widely consumed multi-component frozen product, as a practical indicator for shelf life estimation. The visual appearance of frozen pizzas, particularly the tomato sauce color, plays a key role in how consumers judge them as acceptable or unacceptable during storage [21]. Specifically, the tomato sauce acts as a sensitive indicator of the product’s overall thermal history. Although other components (e.g., the dough or cheese) may remain visually stable for longer at frozen temperatures, consumers associate the perceptible shifts in the red-orange pigment of the sauce with loss of freshness. During storage, one of the main alteration events is the color degradation of the tomato sauce. This event is caused by the oxidation of lycopene, a carotenoid responsible for the red pigmentation in tomatoes, which degrades over time [22]. This degradation occurs even at freezing temperatures, negatively impacting the product’s quality [23]. Quality decay of frozen pizza was monitored through image acquisition during storage at −12 and −18 °C for up to 200 days, and color features were extracted from the tomato sauce region using a dedicated segmentation approach. Consumer acceptability was predicted based on both color features, considering saturation as the main parameter, and storage duration by a logistic regression classifier. By establishing a correlation between color degradation and consumer acceptability, this approach provides a more practical tool for shelf life estimation.

## 2. Materials and Methods

### 2.1. Materials

Freshly made frozen Margherita pizzas, from the same production batch, were provided by a local supplier. Each pizza was individually wrapped in a plastic film and further enclosed in a cardboard box. The ingredients of margherita pizzas were the following: soft wheat flour type ‘0’, mozzarella, water, tomato purée, tomato pulp, sunflower oil, salt, extra virgin olive oil, dextrose, oregano, and yeast.

### 2.2. Methods

#### 2.2.1. Storage Conditions

Packed margherita pizzas were stored under dark conditions in a temperature-controlled freezer set at −12 and −18 °C (±1 °C) for up to 200 days. Temperatures were chosen in order to simulate correct (i.e., −18 °C) and abuse (i.e., −12 °C) conditions to which frozen products are typically subjected during storage. Samples were collected from the freezer at different time intervals for image acquisition and sensory acceptability analysis. The sampling frequency was not fixed in advance, as the kinetics of visual degradation and acceptability changes were initially unknown. Intervals were adaptively determined for each storage temperature based on observed changes to capture relevant quality alterations.

#### 2.2.2. Image Acquisition

A total of 36 frozen pizza samples were imaged during storage using an image acquisition cabinet (Immagini & Computer, Bareggio, Italy) equipped with a digital camera (EOS 550D, Canon, Milano, Italy). The imaging protocol was strictly standardized to eliminate environmental interference, ensure reproducibility and minimize device-dependent variations, which is critical for longitudinal machine learning studies.

To prevent partial thawing or surface structural changes, samples were quickly transferred from storage freezers (−12 or −18 °C) to an imaging cabinet located in a temperature-controlled room maintained below 15 °C. Each imaging session was completed within 50 s to maintain the deep-frozen state, avoiding moisture condensation and preserving the original optical properties of the sample.

The camera was mounted on an adjustable stand at a fixed distance of 80 cm above a black cardboard base, with the optical axis perfectly perpendicular to the sample surface (top-down view) to ensure a consistent imaging angle. Images were captured at the maximum native resolution to provide high-density spatial data for accurate feature extraction. Uniform lighting was provided by four 23 W Philips lamps (Signify, Eindhoven, The Netherlands) (cool daylight, color temperature 6500 K, 230–240 V) disposed at 45°, minimizing shadows, specular highlights, and other artifacts that could compromise color measurements [24].

To ensure that chromatic changes reflected product degradation rather than sensor fluctuations, the camera was operated in full manual mode. Exposure settings were fixed (ISO 200, f/11, 1/50 s) and white balance set at 6500 K. Images were acquired in uncompressed RAW format to preserve maximum color information and avoid the loss of high-frequency spatial data typical of JPEG artifacts.

This standardized protocol was applied consistently across all samples, time points, and storage temperatures, ensuring objective comparisons and maintaining sample integrity throughout the study, providing a robust and objective dataset for the subsequent machine learning pipeline.

#### 2.2.3. Image Preprocessing

Image features were extracted from the 36 original images (one image per frozen pizza sample/time point acquired under standardized conditions). No data augmentation was applied, since the objective was to quantify real, storage-induced visual degradation rather than enlarge the dataset synthetically. The only image operations performed prior to feature extraction were standardization steps (background removal, border/crust exclusion via circular cropping, and HSV-based segmentation of the tomato-sauce region) to ensure that the saturation descriptor reflected the sauce chromatic state consistently across samples.

**Figure 1:**
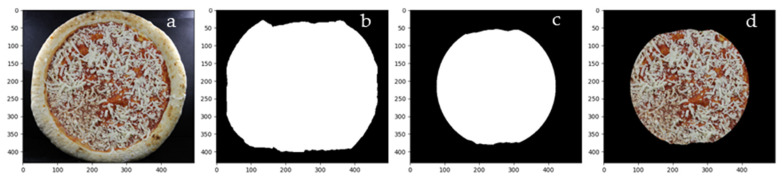
Preprocessing steps applied to frozen pizza images: (**a**) original image, (**b**) initial mask, (**c**) eroded and circular mask, and (**d**) cleaned Image.

To prepare the images for feature extraction, a multi-step pre-processing pipeline was implemented to isolate the internal surface of the pizza and remove unwanted background and structural components. The raw RGB images were first processed using the GrabCut segmentation algorithm [25], which initialized a rectangular region of interest encompassing the pizza and iteratively refined the segmentation mask based on color statistics. The preprocessing steps applied to the frozen pizza images are illustrated in Figure 1. Specifically, Figure 1a shows the original image, Figure 1b the GrabCut-based foreground mask, Figure 1c the refined mask obtained via morphological erosion and circular cropping and finally Figure 1d the cleaned image used for subsequent analysis.

Following segmentation, the mask was refined using morphological erosion to suppress noise and to remove thin or poorly defined borders [26]. To further eliminate edge effects and specifically exclude the crust, which exhibits browning kinetics unrelated to the topping degradation, a circular mask centered on the pizza was applied, intersecting with the eroded output to define a stable, central region of interest. The resulting masked image retained only the core portion of the pizza surface, free of peripheral distortions and spatial inconsistencies.

The final preprocessed image preserves the most homogeneous and visually representative area of the pizza, allowing for reliable extraction of color-based features such as HSV saturation. The final step in the preprocessing pipeline focused on isolating the sauce area based on chromatic characteristics, addressing the challenge of non-uniform topping distribution. Since the tomato-based sauce exhibits a well-defined hue range in the red-orange spectrum, the cleaned RGB images were converted to the HSV color space, which allows for intuitive and independent manipulation of hue, saturation, and brightness components [27].

A dual-thresholding strategy was applied to the hue channel to capture the full range of red tones, including both low-red (0–20°) and high-red (160–180°) intervals. This approach accounted for natural variations in sauce color across different batches and storage durations, as reported in [28]. The resulting binary mask retained only those pixels falling within the defined hue ranges and exhibiting sufficient saturation and brightness levels. Morphological operations, such as opening and closing with a circular structuring element, were subsequently applied to remove noise and small artifacts, improving the coherence of the segmented regions.

The effectiveness of the red sauce segmentation procedure is illustrated in Figure S1. The left panel displays the binary mask generated by the HSV-based thresholding operation, while the right panel shows the corresponding masked image, where only the pixels identified as sauce are retained. Despite the partial occlusion by the mozzarella, the segmentation consistently isolated a sufficient number of pixels to provide a statistically robust representation of the tomato sauce’s chromatic state. The masked result highlights the localized nature of the sauce distribution and confirms that the segmentation procedure effectively isolates the relevant chromatic content from the rest of the pizza surface.

This segmented region forms the basis for the extraction of a suitable color feature, which is used in downstream modeling to estimate visual degradation and predict acceptability.

After image processing, each of the 36 pizza images yielded a 36-feature color descriptor computed on the segmented tomato-sauce region, including the mean, standard deviation, skewness, and kurtosis of the RGB, HSV, and CIELAB channels (9 channels × 4 moments = 36 features). Noise and non-informative variability were reduced by removing the background with GrabCut, refining the ROI mask with morphological filtering (erosion/opening/closing), and excluding border and crust pixels via circular cropping before feature extraction.

#### 2.2.4. Visual Acceptability Analysis

Untrained consumers, recruited among students and workers at the University of Udine and aged between 18 and 64 years, were involved in sensory acceptability tests of frozen Margherita pizza stored for increasing time at −12 and −18 °C. During each test, a random cohort of 60 consumers, with a balanced gender distribution, was involved. Using online surveys, as shown in Figure S2, each consumer was asked to observe a photo of a frozen Margherita pizza sample and answer, based exclusively on its appearance, the following question: “Would you consume the product? Yes or No.”

To identify the specific visual cues that influence consumer decisions, a free-comment section was included. This qualitative step was taken to establish which parameters (e.g., sauce colour, mozzarella appearance or crust texture) were the main focus of consumer judgement. This approach enabled the most relevant chromatic features to be selected for use in subsequent predictive modelling, ensuring the digital analysis remained focused on the attributes most critical to perceived product quality.

The sensory evaluation was conducted via an anonymous online survey. Before participation, all respondents were provided with full disclosure regarding the study’s aims and the requirements of the task. Informed consent was obtained from all participants before they accessed the questionnaire, and they were informed of their right to withdraw at any stage. The study was conducted in strict accordance with the General Data Protection Regulation (GDPR) and the Italian privacy legislation; specifically, the information notice pursuant to Art. 13 of Regulation (EU) 2016/679 was made available to participants through the University of Udine (Uniud) official portal. No personal identifiable information or sensitive data were collected, ensuring the total anonymity of all participants. According to institutional guidelines, formal approval from an ethics committee was not required for this non-invasive, anonymous research involving only the visual assessment of food images.

#### 2.2.5. Statistical Modeling

**Figure 2:**
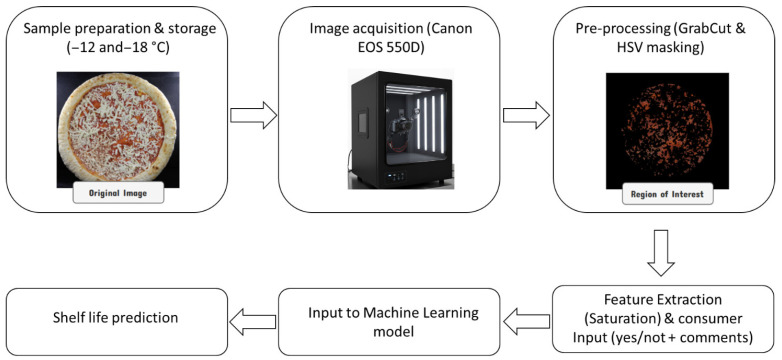
Integrated modelling workflow for shelf-life prediction. The diagram shows how quantitative image features (tomato sauce saturation) and qualitative consumer feedback (e.g., free comments) are combined to calibrate polynomial and logistic regression models.

A correlation analysis was conducted to evaluate the linear association between each color variable, extracted from pizza images stored at −12 and −18 °C, and storage time. The integrated predictive modeling approach (combining feature extraction and consumer validation) is illustrated in Figure 2.

The resulting correlation matrix was visualized as a heatmap, allowing the identification of the color features most strongly correlated with storage time and thereby highlighting their relevance as indicators of pizza degradation. To support the results observed in the heatmap, an average Pearson correlation between all color features extracted and storage time was performed. Consequently, a two-stage supervised modeling approach was employed to characterize sauce color degradation during storage. First, the evolution of saturation over time was modeled via polynomial regression and second, a logistic regression classifier was developed to estimate product acceptability as a function of time and saturation.

##### Polynomial Regression of Tomato Sauce Color Saturation over Time

Given the non-linear pattern of color saturation degradation observed during prolonged frozen storage, saturation as a function of storage time was modeled using a second-degree polynomial regression. This quadratic specification was selected because it is the simplest extension of linear regression that can capture the consistent curvature observed in the saturation trajectories, while remaining parsimonious and readily interpretable. In preliminary model checks, a linear fit produced residual patterns indicative of unmodeled non-linearity, whereas inclusion of the quadratic term reduced this structure and improved fit across samples stored at −12 and −18 °C. The model was fitted by ordinary least squares as Equation (1).

f(t) = β_0_ + β_1_t + β_2_t^2^ (1)

where f(t) denotes the predicted saturation at time t, and β_0_, β_1_, β_2_ are regression coefficients. Model performance was evaluated using standard regression metrics (R^2^ and RMSE), and the contribution of the quadratic term was verified by testing whether β_2_ differed from zero. More flexible machine-learning approaches were not adopted because the primary objective was to provide a compact, physically plausible description of the time-dependent degradation trend rather than maximize predictive performance at the expense of interpretability and increased risk of overfitting for the available dataset.

##### Logistic Regression for Acceptability Classification

To estimate the probability that a product would be perceived as acceptable by consumers, we trained a logistic regression model, a widely used and interpretable approach for binary accept/reject outcomes that directly yields P(acceptable) ∈ [0, 1] and supports effect-size interpretation (e.g., odds ratios), using two predictors: storage time (t, days) and the mean saturation of the segmented tomato-sauce region (S, computed as *HSV_S_mean*). Candidate color descriptors were initially extracted from the sauce region across multiple color spaces (*RGB*, *HSV*, and *CIELAB*), and their relevance to product ageing was screened by correlation analysis with storage time and by consistency across storage conditions (−12 and −18 °C). This screening, together with the qualitative evidence from the consumer survey (≈90% of comments referring to sauce colour changes), identified saturation in HSV as the most informative and stable visual marker; accordingly, *HSV_S_meanHSV\_S\_meanHSV_S_mean* was selected as the single image-derived predictor to avoid redundancy and multicollinearity among highly correlated color variables. The binary response yyy was defined from consumer annotations (“*1*” = acceptable, “*0*” = rejected). The model is expressed as Equation (2).

(2) $$P(y=1|t,S)=\frac{1}{1+exp(-(\alpha_{0}+\alpha_{1}t+\alpha_{2}S))}$$

where *t* is the storage time, *S* is the saturation value, and α0, α1, α2 are the model coefficients. Including both *t* and *S* allows the classifier to jointly account for storage duration and the visual degradation captured by sauce saturation, improving robustness under different freezing temperatures. Model performance was evaluated using accuracy and the area under the ROC curve (AUC).

## 3. Results and Discussion

### 3.1. Choice of Discriminating Parameter for the Consumers

During the online survey, consumers were asked to evaluate the acceptability of Margherita pizzas and provide a comment indicating the main parameter they used to answer the question. From the analysis of these comments, it can be observed that the feature most frequently considered by consumers was the color of the tomato sauce. Approximately 90% of consumers explicitly identified tomato sauce appearance as the primary driver for their response. For illustrative purposes, representative examples of comments provided by consumers on tomato sauce are presented in Table 1. These descriptors directly correlate with the chromatic saturation parameter. Negative comments were attributed to samples with tomato sauce color from orange to yellow, while positive comments were attributed to tomato sauce with bright color and/or strong red.

Although the mozzarella cheese partially obscured the surface, the segmentation pipeline (described in Section 2.2.3) successfully isolated a representative population of sauce pixels. The statistical consistency of these pixels across samples confirms that tomato color serves as a reliable ‘proxy’ for the overall visual degradation of the pizza, as supported by the lycopene oxidation kinetics reported in the literature [23,29,30]. Indeed, due to oxidation, the carotenoids in tomato sauce undergo a loss of redness, especially at frozen temperatures. Therefore, color bleaching from bright red to yellow highlights that the quality of the tomato sauce is deteriorating.

### 3.2. Feature Extraction and Correlation with Storage Time

**Figure 3:**
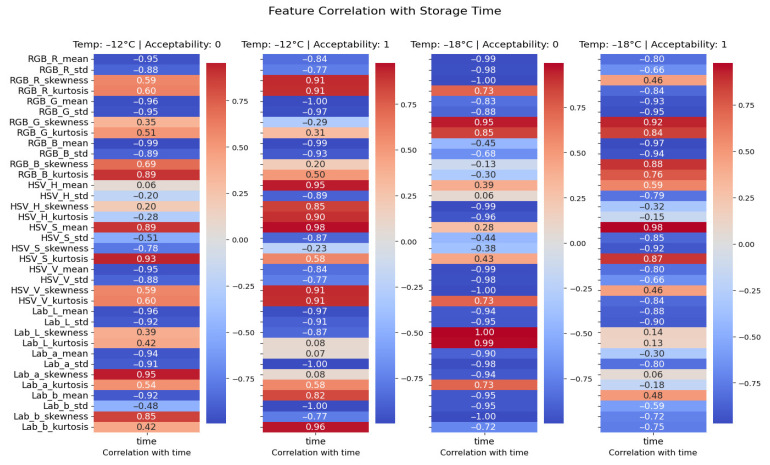
Correlation heatmaps showing the linear relationship between extracted image features and storage time. Each column corresponds to a specific combination of storage temperature and acceptability class.

To further substantiate which color attribute fits better as a predictive feature the correlation analysis was used to evaluate association between each extracted variable and storage time [31]. Figure 3 presents a grid of heatmaps, each representing a specific combination of storage temperature and acceptability class. Among the entire set of color descriptors, including those derived from RGB, HSV, and CIELAB spaces, mean saturation component (*HSV_S_mean*) consistently exhibited a strong positive correlation with time, particularly in acceptable samples stored at both −12 and −18 °C.

In these subsets, the correlation coefficients exceeded 0.98 in magnitude, reflecting a robust and monotonic increase in saturation as storage progressed. This trend was not only stable across temperatures but also aligned with the expected visual degradation observed in the sauce’s appearance, which becomes more vivid and more saturated over time. In contrast, other features, such as *HSV_H_mean* and *HSV_V_mean*, showed either weak or highly variable correlations with time, offering limited predictive value. Features from the RGB and LAB color spaces also exhibited inconsistent behavior across groups, with some descriptors even reversing their correlation signs depending on the acceptability status. From each segmented sauce region, the *HSV_S_mean* was computed as the principal feature. Saturation in the HSV model corresponds to the intensity or purity of the color, ranging from 0 (completely desaturated, i.e., grayscale) to 255 (fully saturated color). Preliminary analyses, similar to work proposed by [32], also confirmed that *HSV_S_mean* exhibited stronger correlation with storage time and panel-based acceptability scores than corresponding features in RGB or LAB spaces. To further evaluate the relevance of the extracted color features with respect to product aging, the average Pearson correlation between each feature and storage time was computed across all experimental groups, including different temperatures and acceptability classes. The results of this analysis are presented in Figure S3. The bar plot displays the average correlation values ranked from highest to lowest, thus offering a global view of how each feature behaves with increasing storage duration. Among all descriptors, *HSV_S_mean* consistently demonstrated the strongest positive correlation with time. Therefore, this color feature was selected as the core input for subsequent modeling steps, due to its ability to capture degradation in visual quality over time in a manner that is both physically meaningful and computationally stable across varying lighting conditions.

### 3.3. Polynomial Regression and Logistic Regression Classifier

The performance of the predictive framework was evaluated by independently assessing both components of the model: the polynomial regression was used to estimate saturation as a function of storage time, and the logistic regression was employed to classify consumer acceptability (Figure S4). Because only 36 independent pizza images were available, we used leave-one-out cross-validation (LOOCV) as the partitioning strategy (35 images for training, 1 for testing at each fold) and calculated accuracy and AUC from the out-of-fold predictions pooled across all 36 folds; all image processing and feature extraction were performed independently per image, and no augmented replicas were generated, so the test image in each fold remained fully unseen during training and data leakage was avoided. A detailed evaluation of these 36 independent predictions revealed that the accuracy of 88.2% corresponds to 32 correct classifications. The four misclassified samples were all located near the 0.5 probability decision threshold. In these borderline cases, visual degradation cues are naturally more subtle, reflecting the transition phase in which consumer perception becomes inherently more uncertain.

The regression model, trained to capture the non-linear degradation trend of sauce saturation over time, demonstrated a high degree of explanatory power. Specifically, the second-degree polynomial fit yielded a coefficient of determination R^2^ of 0.68, indicating that nearly 70% of the variance in saturation values could be explained by time alone. The associated root mean square error (RMSE) was 12.8 units on the *HSV* saturation scale, suggesting acceptable predictive precision across the observed range of storage durations.

In parallel, the logistic regression classifier constructed to estimate the probability of consumer acceptability based on both time and saturation, exhibited strong classification performance. Although the samples were stored at different temperatures (−12 °C and −18 °C), temperature was not included as an independent variable in the logistic regression. Its effect on physical degradation is captured implicitly by the saturation value *HSV_S_mean*, which reflects the actual chromatic state of the sauce and therefore integrates all temperature-mediated oxidative changes. At inference, a user does not need prior knowledge of the storage temperature or duration: the model classifies acceptability from the observed visual state alone. Within the model, storage time (t) serves as a perceptual context variable, it accounts for the temporal dimension of consumer judgment, rather than as a kinetic rate predictor, a role already fulfilled by S. As saturation reflects the actual physical degradation rate, which varies with temperature, it serves as an integrated indicator of the product’s visual state.

The model achieved an overall accuracy of 88.2% when compared to expert-labeled reference data, correctly predicting the acceptability status in the vast majority of cases. To further examine the relationship between saturation and predicted acceptability, the logistic regression model was evaluated by isolating the effect of *HSV_S_mean* at a fixed storage time of 120 days. The probability curve obtained, together with a 95% confidence interval estimated using bootstrap resampling, is shown in Figure S5. The model demonstrates a clear monotonic increase in the probability of acceptability with increasing saturation levels. This trend reinforces the hypothesis that color intensity, as quantified by saturation in the HSV color space, is a key determinant of consumer perception for this product class.

**Figure 4:**
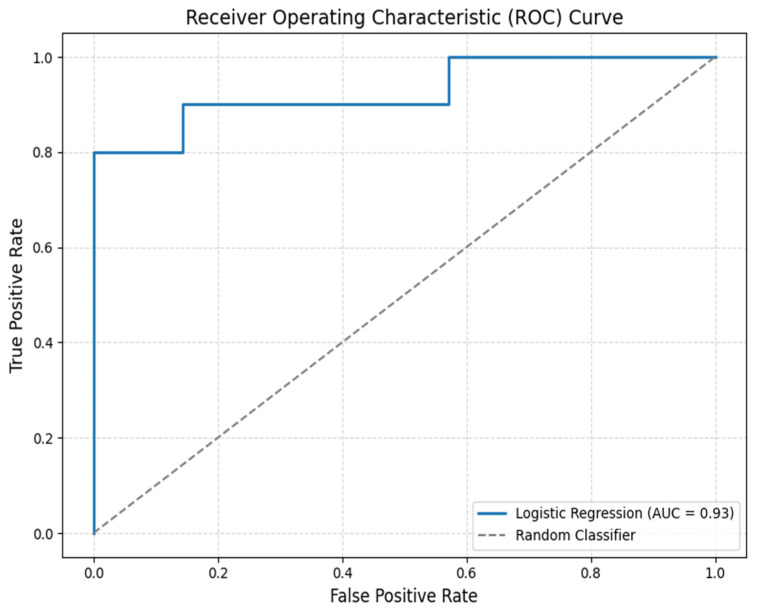
Receiver Operating Characteristic (ROC) curve for the logistic regression model predicting consumer acceptability based on storage time and sauce *HSV_S_mean.* The curve demonstrates the model’s ability to distinguish between acceptable and unacceptable samples across classification thresholds. The area under the curve (AUC = 0.93) confirms the model’s high discriminative performance, substantially outperforming a random classifier.

Additionally, the area under the receiver operating characteristic curve (AUC) was 0.929, reported in Figure 4, confirming the model’s excellent discriminative capacity in distinguishing acceptable from non-acceptable samples based on the provided features. While no standardized metric exists for evaluating the integrated performance of multi-stage predictive frameworks, this study uses the composite confidence score (CCC) [33] to quantify the overall reliability of the proposed acceptability estimation pipeline. The composite confidence score was computed by combining *K* = 3 sub-scores (each scaled to the [0, 1] range) using a weighted average, as reported in Equation (3):

*C* = (*w*1*c*1 + *w*2*c*2 + … + *wK* × *cK*)/(*w*1 + *w*2 + … + *wK*) (3)

where *wK* ≥ 0 represents the relative importance of the k-th component. Because the score is divided by the total weight, the weights do not need to be summed to 1, so a total greater than 1 is not a mathematical contradiction. This approach follows common practice in composite indicators, where bounded components are aggregated, and weights encode their intended relative contribution.

The score consolidates performance indicators from both the regression and classification stages, reflecting their respective contributions to the final decision process. Specifically, the regression model’s coefficient of determination (R^2^) was assigned a weight of 0.68, owing to its foundational role in modeling visual degradation via sauce saturation. The obtained R^2^ means that storage time explains approximately 68% of the variability in the image-derived saturation descriptor, while the remaining 32% reflects sources of variance not captured by time alone. This residual variability is expected in food degradation studies because visual changes depend not only on elapsed time but also on sample-to-sample heterogeneity (e.g., initial sauce formulation and color, topping coverage, local dehydration/oxidation patterns) and measurement noise introduced by imaging conditions and segmentation, even under a standardized acquisition protocol. In other words, storage time is the dominant driver of the trend, but individual pizzas may deviate from the average trajectory due to intrinsic product variability and acquisition/processing uncertainty, which contributes to the unexplained variance.

The classification stage, responsible for mapping saturation and time to consumer acceptability, contributed equally through accuracy and area under the ROC curve (AUC), each weighted at 0.25. This weighting scheme aligns with best practices in composite indicator construction, wherein metric aggregation is guided by the interpretability, influence, and reliability of each component, as discussed by [33] in the *OECD Handbook on Constructing Composite Indicators*.

The resulting CCC value was calculated as 0.75, indicating a high degree of consistency between the model’s explanatory and discriminative capacities. This outcome supports the robustness of the framework and reinforces the potential of image-based, non-destructive methods for acceptability prediction in frozen food products, particularly where visual cues such as color degradation serve as dominant acceptability indicators.

### 3.4. Case Analysis at Fixed Storage Intervals

**Figure 5:**
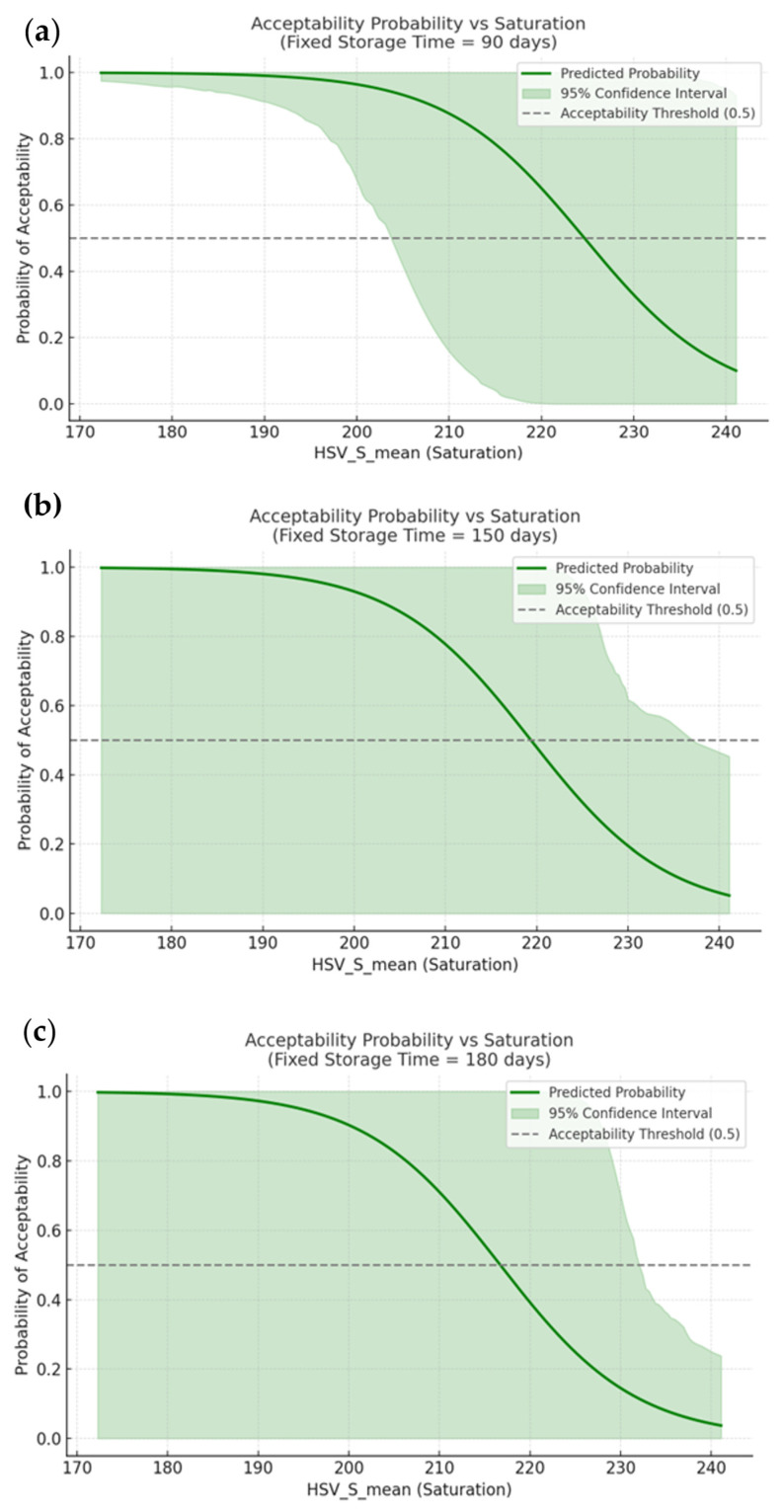
Predicted probability of acceptability as a function of sauce *HSV_S_mean* at fixed storage durations of (**a**) 90, (**b**) 150, and (**c**) 180 days. Each plot includes the logistic model output (green curve) and 95% confidence intervals (shaded area). The horizontal dashed line marks the decision threshold at probability = 0.5.

To provide a more granular understanding of the logistic model’s behaviour across the product’s acceptability, a set of fixed-time simulations was conducted. For each selected time point (i.e., 90, 150, and 180 days), the probability of consumer acceptability was plotted as a function of sauce saturation (*HSV_S_mean*). Each plot includes the model’s predicted probability curve along with a 95% confidence interval estimated via bootstrap resampling (*n* = 1000). As shown in Figure 5, the model maintains a consistent monotonic relationship between saturation and predicted acceptability across time. At earlier time points (e.g., 90 days), the probability curve rises sharply with increasing saturation, and the confidence band is narrow, reflecting high certainty in prediction. As storage time increases, the curve flattens slightly and the confidence interval becomes broader, particularly around the decision threshold at probability 0.5. This widening indicates growing uncertainty in the model’s classification near the end of acceptability, likely due to greater variability in degradation patterns at later stages.

Notably, the point at which the probability crosses the 0.5 threshold shifts toward higher saturation values with time, suggesting that products require a higher visual quality (i.e., stronger saturation) to remain acceptable in later storage stages. In particular, when the time is fixed at 90 days, the acceptability limit, expressed as a saturation value, is about 225. However, at 150 and 180 days is about 220 and 216, respectively. This trend aligns with empirical expectations and supports the dynamic interpretation of acceptability based on color degradation.

Several studies have been conducted in the literature to evaluate product quality, correlating the use of ML with image analysis techniques to predict the shelf life of perishable products [15,16,17]. The cited studies showed that the characteristics extracted from the images can be considered strong and reliable indicators for estimating shelf life using machine learning. However, these results were obtained by consulting the integrity of the product (absence of physical defects or surface damage) rather than the consumer opinion. Therefore, in the present study, by combining consumer acceptability with an image-based approach, it was possible to develop a machine learning model able to predict acceptability limits, based on both saturation and storage duration, corresponding to a saturation value of the tomato sauce on frozen pizza. Although in-person sensory sessions offer a holistic experience, including attributes such as aroma and texture, the use of standardized, high-resolution images in online consumer surveys has distinct advantages in terms of statistical power and scalability [34]. By presenting the same digital stimulus to a large group of people (*n* = 60 each time and storage temperatures), we eliminated the variability caused by products thawing during traditional sessions. In fact, conducting visual evaluations of deep-frozen products in person presents significant methodological challenges. The rapid transition from storage at −18 °C to room temperature triggers immediate surface changes, such as melting frost and moisture condensation. These changes would inevitably bias the consumer’s perception of colour and freshness.

Furthermore, this approach aligns with modern e-commerce trends, in which visual acceptability of digital previews is the primary driver of consumers’ initial purchasing decisions.

## 4. Conclusions

This study presents a machine learning model for predicting the acceptability of frozen pizza. The model focuses on visual degradation of the tomato-based sauce as a key indicator of quality. The mean saturation value in the HSV colour space was found to be the most reliable indicator of visual freshness and pizza degradation, serving as a proxy for lycopene oxidation. A key finding of this research is the dynamic shift in consumer perception. The results demonstrate that the longer the storage time, the more stringent the acceptability threshold becomes. This suggests that consumer expectations evolve over the frozen product’s storage. However, some limitations must be acknowledged. This proof of concept focused exclusively on visual acceptability, which, although the main reason for rejecting frozen foods, does not take into account other sensory attributes such as taste and sniff. Furthermore, although using colour saturation as a non-destructive physical proxy for chemical degradation is effective for rapid industrial monitoring, direct chemical analysis is still necessary for a more in-depth understanding. These findings demonstrate the potential for the frozen food industry to replace traditional, destructive shelf life testing with real-time, sensor-based monitoring. To enhance the robustness of this framework, future research should integrate multi-batch variability and real-world storage scenarios to improve the model’s performance in industrial settings and to account for diverse domestic storage conditions.

## Figures and Tables

**Table 1:** Representative consumer comments regarding pizza acceptability, highlighting the influence of tomato sauce color on their evaluations. Comments are divided into positive and negative acceptability responses.

Acceptability Comments	
Positive	Negative
Tomato sauce has a nice red color	Tomato sauce has an orange color
Tomato sauce has a brilliant color	The tomato sauce appears faded
Tomato sauce has an acceptable red color	Tomato sauce has a yellow color
Tomato sauce has a uniform red color	Tomato sauce has a non-uniform color (yellow or orange)

## Data Availability

The original contributions presented in this study are included in the article/Supplementary Materials. Further inquiries can be directed to the corresponding author.

## Supplementary Materials

The following supporting information can be downloaded at: https://www.mdpi.com/article/10.3390/foods15081348/s1, Figure S1: Segmentation of the sauce-covered region using HSV color thresholding: (a) binary mask representing areas identified as red-orange sauce; (b) masked image displaying only the sauce pixels retained after thresholding and morphological filtering; Figure S2: Illustrative example of question asked to consumers in the online survey; Figure S3: Average Pearson correlation between each extracted feature and storage time, computed across all temperature and acceptability groups; Figure S4: Logistic regression prediction surface showing the probability of consumer acceptability as a function of storage time and *HSV_S_mean* for the merged T–12 °C and T–18 °C storage group. The background color gradient represents the predicted probability of acceptability, ranging from low (red) to high (green). The black dashed contour indicates the decision boundary at a probability threshold of 0.5. Observed samples are overlaid, with across markers indicating samples classified as acceptable and cross markers indicating non-acceptable samples, according to the expert-labeled ground truth; Figure S5: Predicted probability of consumer acceptability as a function of sauce *HSV_S_mean*, at a fixed storage time of 120 days. The green curve represents the logistic regression model output, while the shaded area denotes the 95% confidence interval obtained from bootstrap resampling. The dashed horizontal line marks the acceptability threshold at 0.5. The curve highlights a strong and statistically robust relationship between saturation and predicted acceptability.
