# Supplementary material for: Image-Based Machine Learning for Predicting Acceptability Limits in Frozen Pizza Shelf Life
Source: Foods. 2026 Apr 13;15(8):1348. doi: 10.3390/foods15081348 (PMC13115155; doi:10.3390/foods15081348)
Supplement: Supplementary file 1 [file foods-15-01348-s001.zip › foods-4177606-supplementary.pdf]

Supplementary Materials

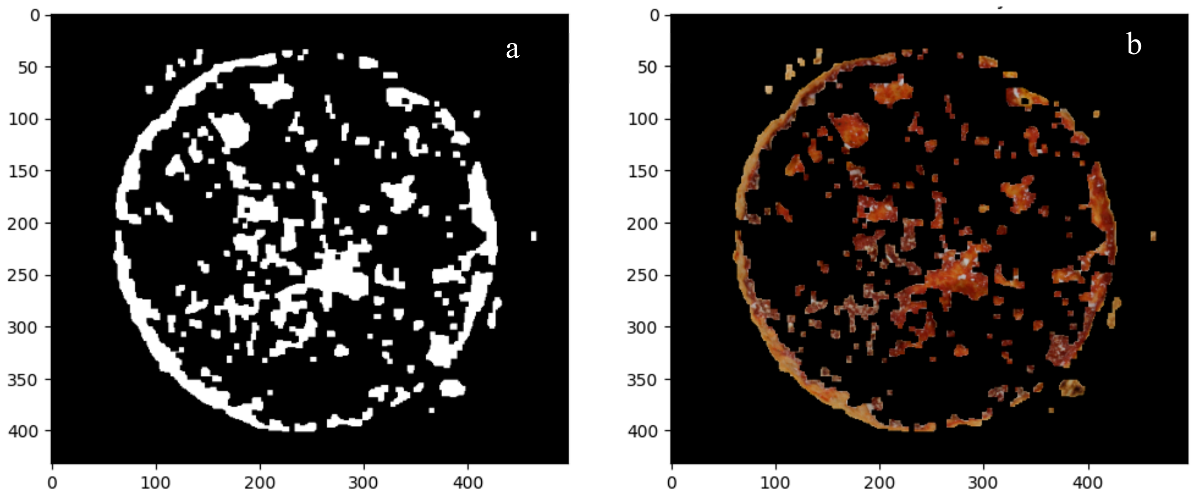

Figure S1. Segmentation of the sauce-covered region using HSV color thresholding: a) binary mask representing areas identified as red-orange sauce; b) masked image displaying only the sauce pixels retained after thresholding and morphological filtering.

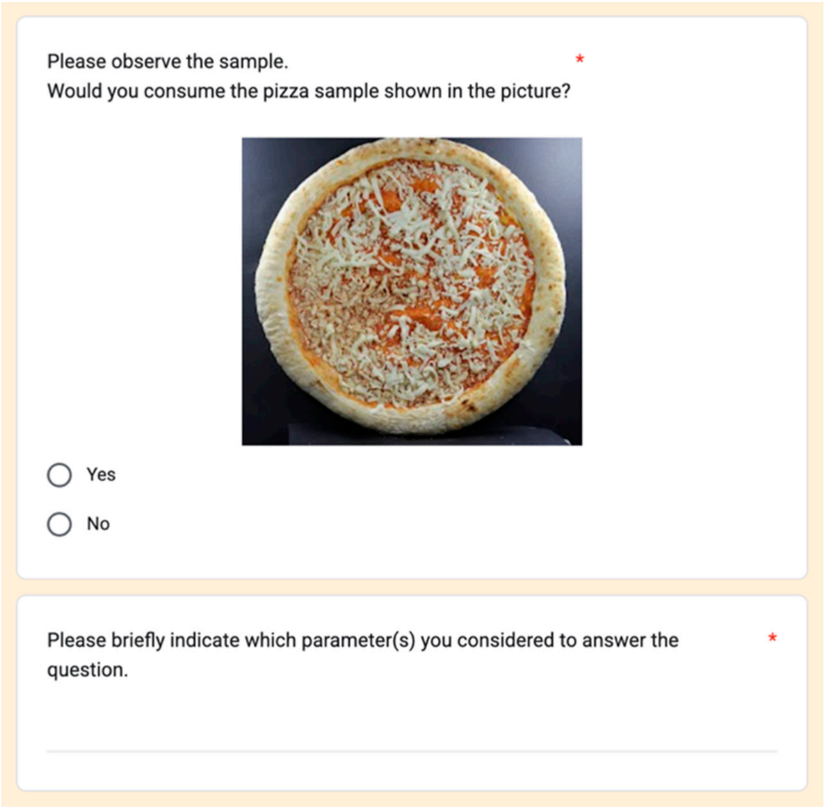

Figure S2. Illustrative example of question asked to consumers in the online survey.

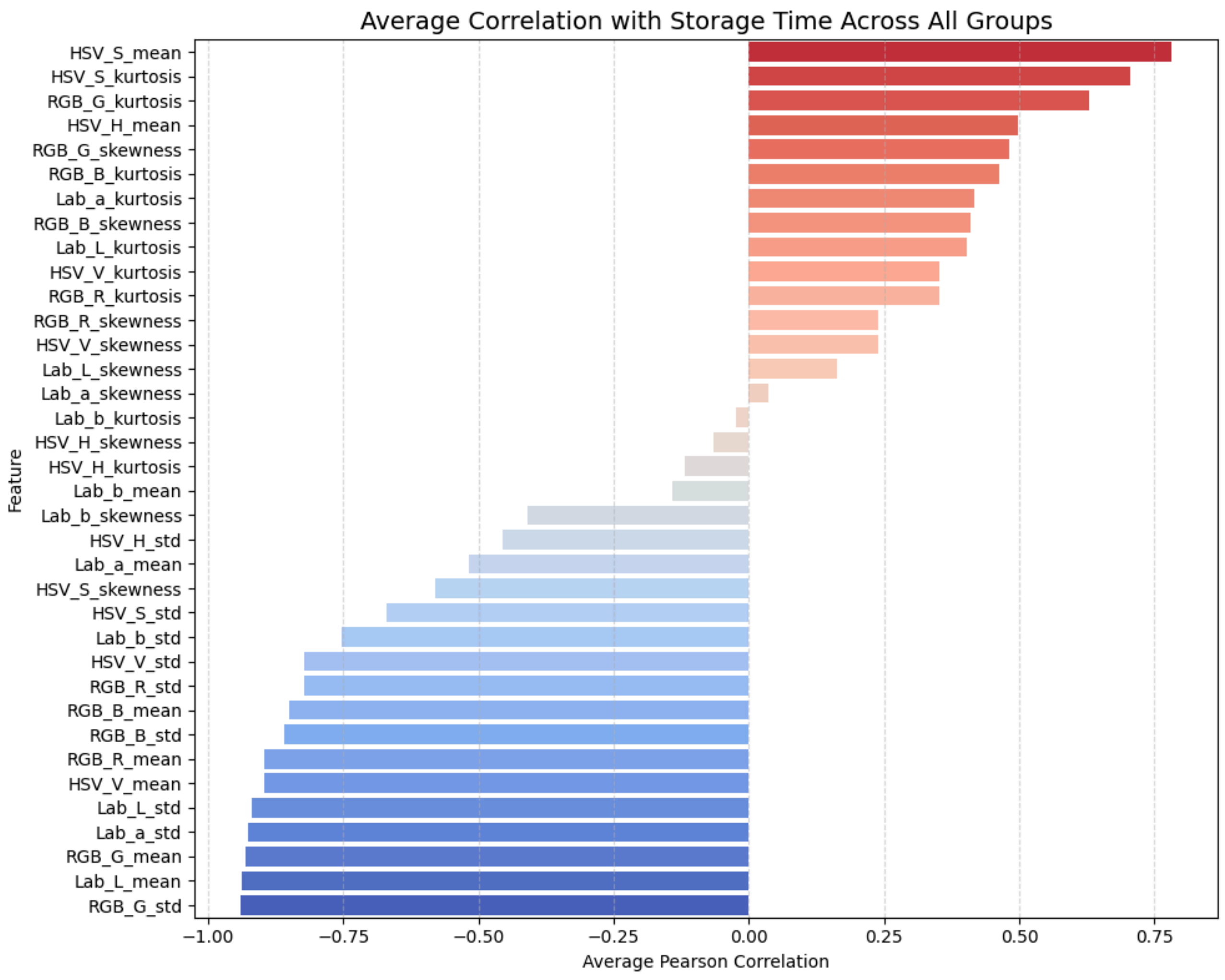

Figure S3. Average Pearson correlation between each extracted feature and storage time, computed across all temperature and acceptability groups.

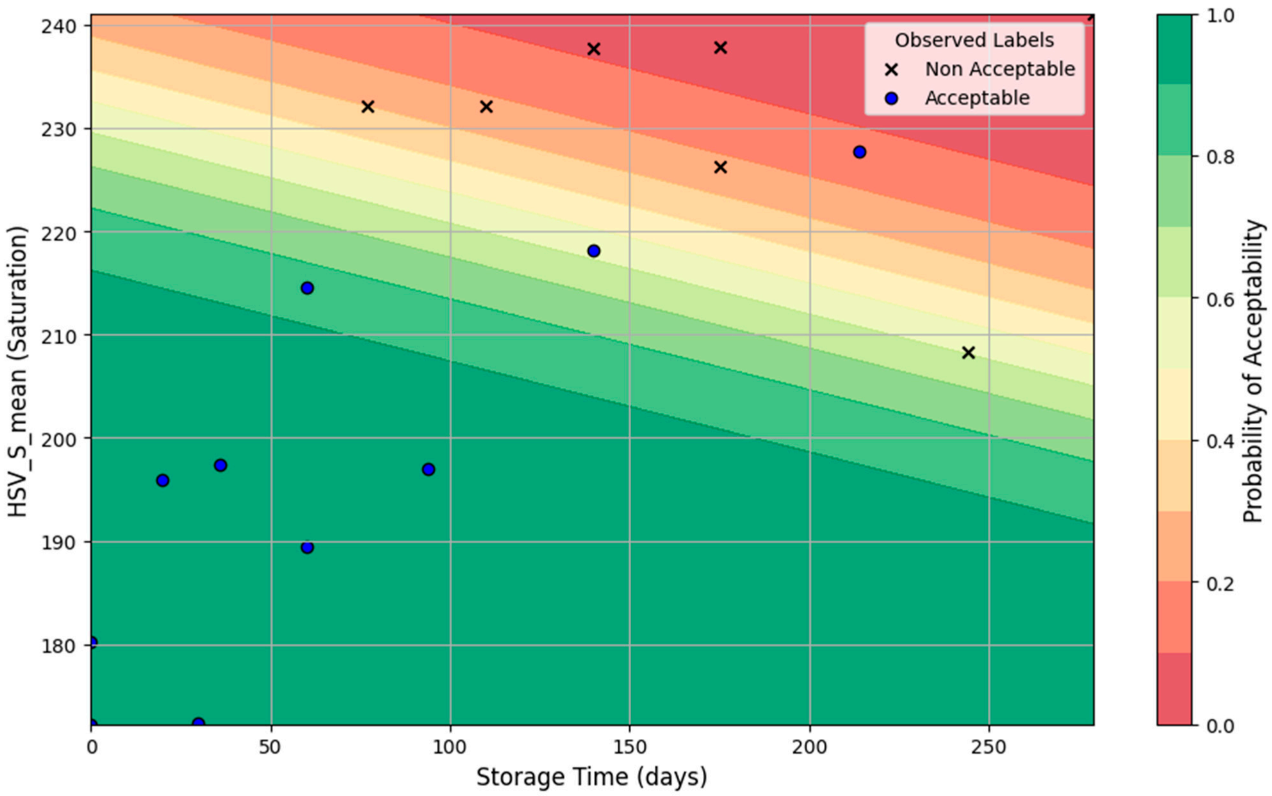

Figure S4. Logistic regression prediction surface showing the probability of consumer acceptability as a function of storage time and *HSV_S_mean* for the merged T–12 °C and T–18 °C storage group. The background color gradient represents the predicted probability of acceptability, ranging from low (red) to high (green). The black dashed contour indicates the decision boundary at a probability threshold of 0.5. Observed samples are overlaid, with across markers indicating samples classified as acceptable and cross markers indicating non-acceptable samples, according to the expert-labeled ground truth.

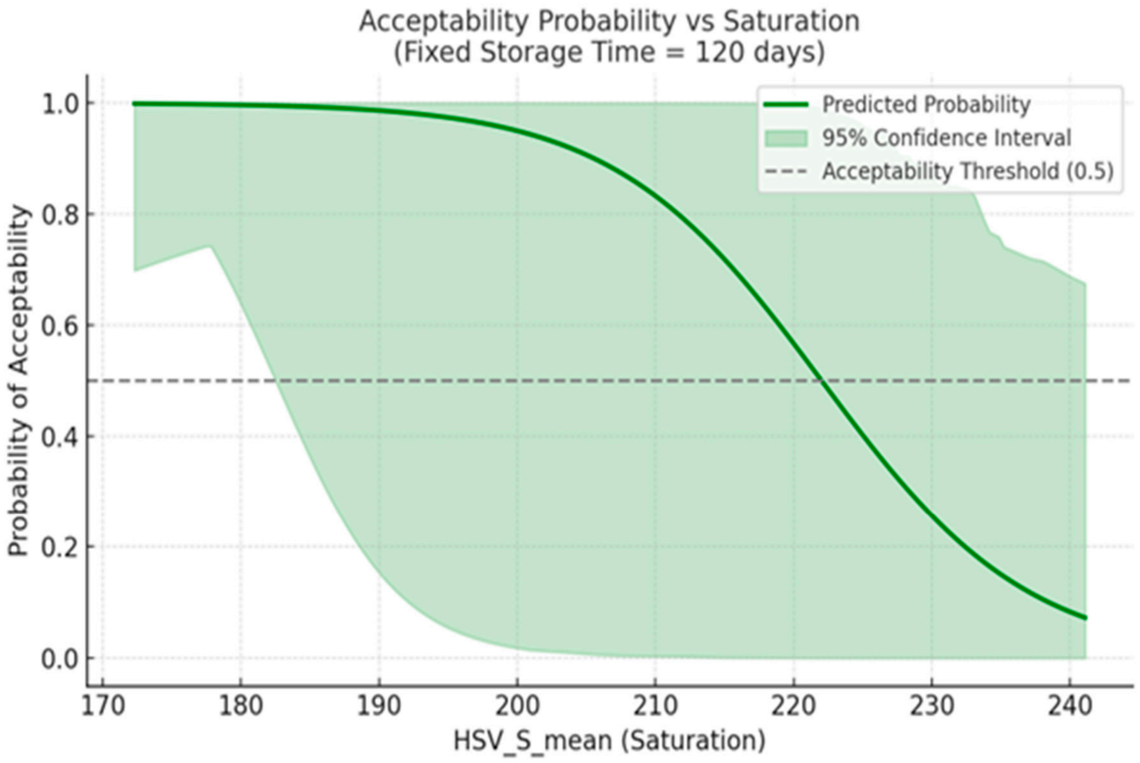

Figure S5. Predicted probability of consumer acceptability as a function of sauce *HSV_S_mean*, at a fixed storage time of 120 days. The green curve represents the logistic regression model output, while the shaded area denotes the 95% confidence interval obtained from bootstrap resampling. The dashed horizontal line marks the acceptability threshold at 0.5. The curve highlights a strong and statistically robust relationship between saturation and predicted acceptability.
